# Long-Term Analyses of SARS-CoV-2 Humoral and T Cell Responses and Breakthrough SARS-CoV-2 Infections after Two Doses of BNT162b2 Followed by mRNA-1273 and Bivalent Omicron-Adapted BNT162b2 Vaccines: A Prospective Study over 2 Years in Non-Immunocompromised Individuals

**DOI:** 10.3390/vaccines11121835

**Published:** 2023-12-10

**Authors:** Alejo Erice, Lola Prieto, Cristina Caballero

**Affiliations:** 1 Department of Internal Medicine, Hospital Asepeyo, 28823 Coslada, Spain; 2 Unidad de Apoyo a la Investigación, Facultad de Medicina, Universidad Francisco de Vitoria, 28223 Pozuelo de Alarcón, Spain; lola.prieto@ufv.es (L.P.); ccaballerogarcia@asepeyo.es (C.C.); 3 Clinical Diagnostic Laboratory, Hospital Asepeyo, 28823 Coslada, Spain

**Keywords:** SARS-CoV-2, SARS-CoV-2 vaccine, BNT162b2, mRNA-1273, bivalent Omicron-adapted vaccine, SARS-CoV-2 antibody humoral immune response, SARS-CoV-2 T cell response, immune imprinting

## Abstract

Long-term analyses of the immune response following SARS-CoV-2 mRNA vaccines are essential to determining its characteristics and providing the basis for vaccination strategies. We conducted a prospective study in a cohort of 268 healthy adults followed for >2 years after two doses of BNT162b2. Antibodies targeting the receptor-binding domain of the S1 subunit of the spike of SARS-CoV-2 (anti-RBD) were measured at eight time points; T cell response was analyzed using an interferon-γ release assay. A total of 248 (93%) subjects received mRNA-1273 on month 9; 93 (35%) received the bivalent Omicron-adapted BNT162b2 vaccine between months 19 and 26. Breakthrough infections occurred in 215 (80%) participants, with frequencies unaffected by the additional vaccines. Anti-RBD declined over the initial 9 months, increased after mRNA-1273, and declined gradually thereafter. In 50 (17%) previously infected subjects, anti-RBD levels were significantly higher up to month 9 (*p* < 0.05) but subsequently declined below those of uninfected individuals. Anti-RBD titers protective against SARS-CoV-2 could not be defined. Most subjects developed a positive T cell response that remained after 26 months. Waning of protection against SARS-CoV-2 infection occurred over time, resulting in non-severe breakthrough infections in most participants. The evolution of anti-RBD suggests modulation of the immune response through immune imprinting.

## 1. Introduction

In a phase 3 clinical trial conducted before the emergence of severe acute respiratory syndrome coronavirus 2 (SARS-CoV-2) variants Delta (B.1.617.2) and Omicron (B.1.1.529), two doses of the mRNA vaccine BNT162b2 (Comirnaty, BioNTech Manufacturing GmbH, Mainz, Germany) were 95% protective against symptomatic infection [1]. On longer-term follow-up, the vaccine efficacy decreased to 84% within 7 months [2,3], due to waning immunity and the emergence of variants with the capacity to evade vaccine-induced protection [4,5]. This led to the recommendation of administering a third mRNA vaccine dose (booster) [6], and, later, an additional dose of Omicron-adapted vaccines [7,8].

Numerous studies have described the efficacy of booster and bivalent mRNA vaccines in preventing severe SARS-CoV-2 infections, hospitalizations, and deaths; however, long-term follow-up data on the humoral and cell-mediated immune responses following repeated SARS-CoV-2 vaccinations are limited. Therefore, analyses of the immune response over extended periods of time following the administration of SARS-CoV-2 mRNA vaccines help to define its characteristics and durability; together with clinical efficacy studies, those analyses contribute to the basis for specific recommendations about the need for additional vaccine doses.

We described an early decline of vaccine-induced antibodies after the first two doses of BNT162b2 in a cohort of non-immunocompromised subjects [9]. We now extend our observations up to >2 years after the second dose of BNT162b2 in the same cohort of individuals, some of whom also received a booster dose of mRNA-1273 (Spikevax, ModernaTx, Inc., Norwood, MA, USA) and later, a dose of the Pfizer-BioNTech (Mainz, Germany) bivalent Omicron-adapted BNT162b2 mRNA vaccine. Vaccine-elicited T cell-mediated response was also analyzed.

We found that vaccine-induced anti-RBD antibody titers declined after a temporary increase following mRNA-273 and did not increase after the bivalent Omicron-adapted BNT162b2 mRNA vaccine. Specific protective anti-RBD antibody titers against SARS-CoV-2 infection could not be defined. Waning of immune protection occurred, resulting in non-severe breakthrough infections in most study participants due to dominance of immune-evading variants in the presence of a durable vaccine-induced SARS-CoV-2-specific T cell response.

## 2. Materials and Methods

### 2.1. Study Design

This prospective longitudinal observational study was conducted at a single institution (Hospital Asepeyo, Coslada, Madrid, Spain) between March 2021 and April 2023. Non-immunocompromised hospital workers >18 years of age who received two doses of BNT162b2 between January and March 2021 were eligible to participate. Blood samples were collected from study participants at eight different time points after the second dose of BNT162b2. Study participants were offered a dose of the mRNA-1273 vaccine in December 2021 and the bivalent Omicron-adapted BNT162b2 vaccine in October 2022.

### 2.2. Anti-SARS-CoV-2 Antibodies

Antibodies against the receptor-binding domain of the S1 subunit of the spike protein of SARS-CoV-2 (anti-RBD antibodies) were measured at all time points using a quantitative immunoassay (Architect SARS-CoV-2 IgG II Quant, Abbott, Chicago, IL, USA). Results ≥ 50 arbitrary units (AU)/mL were considered positive. Antibodies targeting the SARS-CoV-2 nucleocapsid (anti-N antibodies) were measured using a qualitative immunoassay (Architect SARS-CoV-2 IgG, Abbott); cut-off index values ≥ 0.49 were considered positive.

### 2.3. T Cell Response

SARS-CoV-2-specific T cell response was analyzed at two time points using an interferon gamma (IFN-γ) release assay (Quant-T cell SARS-CoV-2 + Quan-T cell ELISA, EUROIMMUN, Lübeck, Germany). Heparinized whole blood was incubated for 24 h in tubes coated with antigens based on the S1 domain of the spike protein of SARS-CoV-2. Following incubation, plasma was separated by centrifugation, and IFN-γ was quantified by ELISA. Because the number of T lymphocytes in each sample was not determined, there was no correlation between the concentration of IFN-γ and the magnitude of the T cell response. Controls were included in each run to confirm that the number of T lymphocytes per sample was appropriate to provide a valid result. Results > 200 mUI/mL of IFN-γ were considered positive.

### 2.4. SARS-CoV-2 Infections

At each study time point, participants filled a questionnaire that collected information on the development of symptoms of SARS-CoV-2 infection since the previous blood sampling and the results of the diagnostic tests performed. Symptomatic SARS-CoV-2 infections were defined by the presence of symptoms together with a positive polymerase chain reaction or antigen test for SARS-CoV-2 in upper respiratory tract samples. Asymptomatic SARS-CoV-2 infections were diagnosed by the de novo appearance of anti-N antibodies and/or a >5.1% increase in titers of anti-RBD antibodies [10]. Reinfections were considered in subjects with previous infections showing positivization or a >5.9% increase in titers of anti-N antibodies, and/or a titer increase >5.1% of anti-RBD antibodies [10] not justified by a booster or bivalent vaccine administered within the previous three months.

Information on the dominant SARS-CoV-2 variants during the study period was obtained from the official web page on COVID-19 of the Spanish Ministry of Health [11].

### 2.5. Statistical Analysis

Changes in anti-RBD antibody titers over time were analyzed using a linear mixed model with random intercept. The model was adjusted for age, gender, previous documented SARS-CoV-2 infection, SARS-CoV-2 infection during follow-up, time elapsed since the last SARS-CoV-2 vaccination, presence of an immune-conferring event (vaccination or infection), and number of SARS-CoV-2 infections/reinfections during follow-up. Anti-RBD antibody titers were log-transformed and presented as geometric mean titer (GMT) ratios with a 95% confidence interval. The model included 258 participants because information on previous documented SARS-CoV-2 was unavailable for 10 subjects. Results are presented, disaggregated by gender (male/female), age (<47 years, ≥47 years), previous documented SARS-CoV-2 infection prior (yes/no), and SARS-CoV-2 infection during follow-up (yes/no). The Bonferroni correction method (pairwise comparisons) was used to compare anti-RBD antibody titers over time and between groups. The effect of mRNA-1273 and Omicron-adapted bivalent BNT162b2 vaccines on the occurrence of subsequent SARS-CoV-2 infections/reinfections was analyzed using Chi-square or Fischer’s exact tests. Differences in the proportion of participants with a positive T cell response were assessed using McNemar’s test for paired data. Statistical analyses were performed using R 3.6 (R Project for statistical computing) and STATA 15.0 (Stata Corporation, College Station, TX, USA). All *p*-values were 2-sided; a *p*-value < 0.05 was considered significant.

## 3. Results

### 3.1. Study Population

Two-hundred and seventy-three subjects received two doses of BNT162b2 at our institution between December and January of 2021. Of those, 270 enrolled in the study; 2 were excluded from the analyses due to immunocompromised conditions. The mean age of the remaining 268 participants was 46 years (SD 11.5); 167 (62%) were female and 101 (38%) were male.

**Figure 1:**
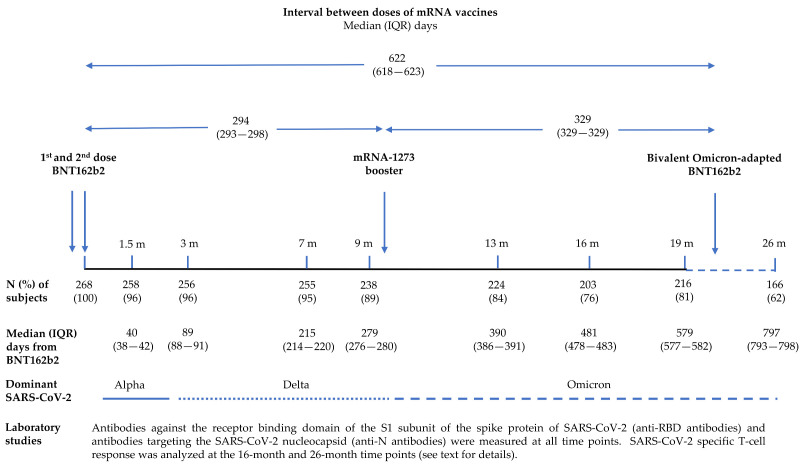
Study design.

A total of 1816 blood samples were collected from study participants 1.5, 3, 7, 9, 13, 16, 19, and 26 months after the second dose of BNT162b2. Two-hundred and forty-eight (93%) subjects received the mRNA-1273 booster between December 2021 and January 2022, immediately after the 9-month blood extraction; 93 (35%) participants received the bivalent Omicron-adapted BNT162b2 vaccine in October–December 2022, between the 19-month and 26-month blood extractions. The study design is shown in Figure 1.

Overall, 89 (33%) subjects received all four vaccine doses, 248 (93%) received two doses of BNT162b2 plus the mRNA-1273 booster, and 4 (1%) received two doses of BNT162b2 plus the bivalent Omicron-adapted BNT162b2 vaccine.

### 3.2. SARS-CoV-2 Infections during the Study Follow-Up

Dominant SARS-CoV-2 variants during the study were Alpha (B.1.1.7) when the two doses of BNT162b2 were administered and at the time of the 1.5-month blood extraction, Delta (B.1.617.2) at the times of the 3- and 9-month blood extractions, and Omicron (B.1.1.529) and its subvariants at the times of the 13-, 16-, 19-, and 26-month blood extractions [11].

During the study follow-up, 215 (80%) participants had 345 episodes of documented SARS-CoV-2 infections (Table 1). Thirty-seven (11%) infections occurred prior to the first dose of BNT162b2, 36 (10%) between the administrations of the second dose of BNT162b2 and the mRNA-1273 booster, 209 (60.5%) between the mRNA-1273 booster and the bivalent Omicron-adapted BNT162b2 vaccine, and 63 (18%) after the bivalent Omicron-adapted BNT162b2 vaccine. One hundred and seventy-one (50%) of the infections were symptomatic, with one (0.6%) requiring hospitalization; 174 (50%) were asymptomatic. Two hundred and seventeen (63%) were primary infections, and 128 (37%) were reinfections (as defined earlier). Fifty-three (20%) individuals remained uninfected for the entire duration of the study.

Information on SARS-CoV-2 breakthrough infections occurring after the mRNA-1273 booster and prior to the Omicron-adapted mRNA vaccine (obtained at the 13-month, 16-month and 19-month time points) was available for 248 (92.5%) study participants; similarly, information on SARS-CoV-2 breakthrough infections occurring after the bivalent Omicron-adapted mRNA vaccine (obtained at the 26-month time point) was available for 166 (62%) study participants. Frequencies of SARS-CoV-2 breakthrough infections/reinfections were not significantly different among participants who received the mRNA-1273 booster or the bivalent Omicron-adapted BNT162b2 vaccine and those who did not receive the additional vaccine doses (Table 2).

### 3.3. Kinetics of SARS-CoV-2 Anti-RBD Antibodies

**Figure 2:**
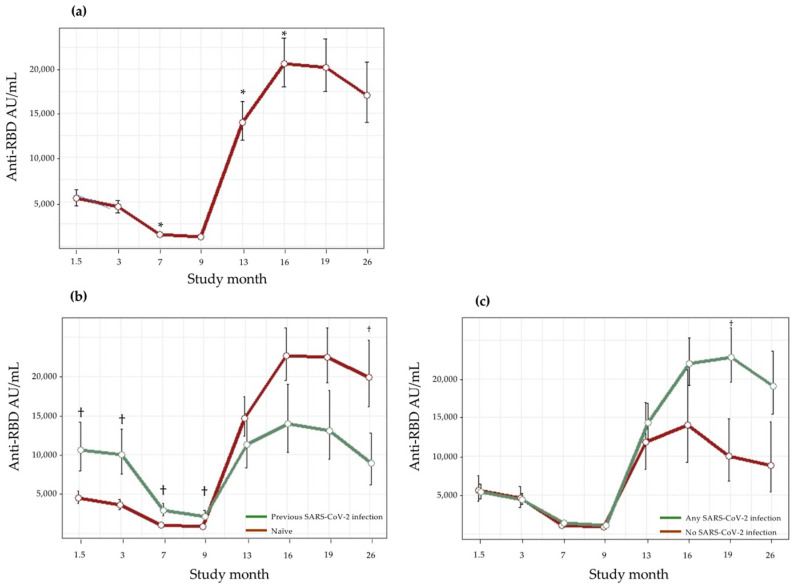
Evolution of anti-RBD antibodies during follow-up. (**a**) Anti-RBD antibodies in all study participants; (**b**) Anti-RBD antibodies in individuals infected and uninfected (naïve) before the first dose of BNT162b2 or prior to the 1.5-month time point; (**c**) Anti-RBD antibodies in subjects acquiring SARS-CoV-2 infection anytime during follow-up and in naïve subjects who remained uninfected throughout the entire study (see text for additional details). * Significant difference (*p* < 0.05) compared with the previous time point; ^†^ Significant difference (*p* < 0.05) among groups at indicated time points.

The evolution of anti-RBD antibody titers during the study period according to age, gender, and SARS-CoV-2 infection status at baseline and during follow-up is shown in Table 3 and Figure 2. Males (compared to females) and <47-year-old participants (compared to ≥47-year-olds) had higher anti-RBD antibody titers at all study time points, although the differences were not statistically significant.

Anti-RBD antibodies declined progressively over the initial 9 months after the administration of the second dose of BNT162b2. Subsequently, there was a sharp increase in titers at the 13-month time point, shortly after the administration of the mRNA-1273 booster to 248 (93%) of study participants; this was followed by a moderate increase in titers at the 16-month time point. Thereafter, anti-RBD antibodies declined progressively at the 19- and 26-month time points; 93 (35%) of the study participants received the Omicron-adapted BNT162b2 vaccine between those two final time points.

In the 50 (17%) individuals who were previously infected by an ancestral SARS-CoV-2 variant, either before the first dose of BNT162b2 (*n* = 37) or prior to the 1.5-month time point (*n* = 13), anti-RBD antibody titers were significantly higher as compared to those in uninfected (naïve) subjects up to the 9-month time point (*p* < 0.05); thereafter, anti-RBD titers in previously infected subjects fell to below those in naïve individuals at all remaining time points (*p* < 0.05 at the 26-month time point).

In the 53 (20%) naïve subjects who also remained uninfected throughout the entire study, anti-RBD antibody titers were initially similar to those in subjects who acquired SARS-CoV-2 infections at any time during the study; however, from the 7-month time point to the end of the study, their anti-RBD antibody titers were lower.

### 3.4. SARS-CoV-2-Specific T Cell Response

SARS-CoV-2-specific T cell response was determined in blood samples collected at the 16-month and 26-month time points from 190 (71%) and 166 (62%) study participants, respectively. At the 16-month time point, 184 (97%) subjects had a positive T cell response, whereas a specific T cell response was not detected in 6 (3%) individuals. At the 26-month time point, 154 (93%) subjects had a positive T cell response, whereas a specific T cell response was not detected in 12 (7%) individuals. T cell response was measured at both time points in 142 (53%) subjects: 127 (89%) had a positive result both times, 9 (6%) had an initial positive result followed by a negative result, and 6 (4%) had an initial negative result followed by a positive result. Overall, the frequencies of a positive or a negative SARS-CoV-2-specific T cell response at the 16-month and 26-month time points were not statistically significant.

## 4. Discussion

In this study of healthy adults who received two doses of BNT162b2, a booster with mRNA-273, and a bivalent Omicron-adapted BNT162b2 vaccine over a 26-month period, the evolution of anti-RBD antibody titers showed three distinct phases. The first phase, spanning the first 9 months after the second dose of BNT162b2, was characterized by a progressive decrease in anti-RBD antibodies. As observed in other studies, anti-RBD antibody titers were higher at all time points in subjects previously infected with an ancestral SARS-CoV-2 variant [12,13,14,15,16]. The few breakthrough infections that occurred in this phase were most likely the consequence of waning of vaccine-induced protection and the dominance of the Delta (B.1.167.2) variant, with its increased replicating fitness and evasiveness from BNT162b2-induced neutralizing antibodies [17,18].

The second phase, extending from 9 to 16 months after the second dose of BNT162b2, was characterized by an overall increase in anti-RBD antibody titers and a rise in breakthrough SARS-CoV-2 infections coincident with Omicron (B.1.1.529) dominance. The initial increase observed at 13 months most likely represented the residual effect of the mRNA-1273 booster (administered to 93% of the study participants after the 9-month time point), whereas the subsequent increase observed at 16 months was the consequence of breakthrough SARS-CoV-2 infections. This observation mirrors the results reported in a study among healthcare workers after an almost identical triple-vaccination schedule [19].

The third phase, from 16 to 26 months after the second dose of BNT162b2, showed a decline in anti-RBD antibody titers together with ongoing SARS-CoV-2 breakthrough infections (also during Omicron (B.1.1.529 dominance). The decline in anti-RBD antibodies was significantly more pronounced in subjects who had been infected with an ancestral SARS-CoV-2 variant prior to the 1.5-month time point. Most of the SARS-CoV-2 breakthrough infections of the study occurred during this period, indicating waning immune protection and evasiveness of the dominant circulating variant (Omicron B.1.1.529). Of note is that 35% of the study participants received the bivalent Omicron-adapted BNT162b2 vaccine in this phase. Clinical studies have shown a reduced risk of symptomatic and severe SARS-CoV-2 infections associated with bivalent Omicron-adapted vaccines that wanes over three months; however, data on the evolution of anti-RBD titers following its administration are very limited [17,20,21].

The kinetics of the anti-RBD antibody response during the third phase of the study (from 16 to 26 months after the second dose of BNT162b2) suggest immune imprinting. The overall decline of anti-RBD antibodies in this phase and the more pronounced decline of anti-RBD antibodies in individuals previously infected by an ancestral SARS-CoV-2 variant (as compared to naïve individuals) could reflect modulation of the humoral response against the antigenic RBD domain of new variants [22,23]. In addition, hybrid immune damping could have occurred as a consequence of repeated antigenic exposures of vaccinated subjects through SARS-CoV-2 breakthrough infections and/or the administration of the mRNA-1273 booster and/or the Omicron-adapted bivalent BNT162b2 vaccine [24].

A SARS-CoV-2-specific T cell response was detectable in 97% of our study participants more than 9 months after two doses of BNT-162b2, when most had not yet been infected; this durable T cell response is in contrast with the substantial decline in anti-RBD antibodies that occurred during the same period. A SARS-CoV-2-specific T cell response was also detectable in 94% of the subjects 26 months later; because most participants had been infected by that time point, the T cell response could be the consequence of prior vaccination and/or additional doses of vaccines and/or breakthrough infections during the dominance of Delta (B.1.617.2) and Omicron (B.1.1.529) and its subvariants. Most of the breakthrough infections were not severe, and 50% were asymptomatic, highlighting the lasting and broadly cross-reactive cell-mediated immune response protecting against severe disease induced by prior vaccination and/or infection [25,26].

Titers of vaccine-induced antibodies that are protective against SARS-CoV-2 infection have not been established. In previous studies of anti-RBD antibodies, a threshold of 4160 AU/mL was used as the surrogate marker for neutralizing activity [27]; in addition, a threshold of 3563 AU/mL [equivalent to 506 binding antibody units (BAU)/mL using the WHO international standard] was also used as the surrogate marker for protection against SARS-CoV-2 infection [28]; this threshold has been correlated with a vaccine efficacy of 80% against most pre-Omicron variants [29]. In our study, anti-RBD antibody titers in the group of subjects who remained uninfected throughout the entire follow up were above those thresholds most of the time but remained lower than those in participants who acquired breakthrough infections. Therefore, titers of anti-RBD antibodies that are protective against SARS-CoV-2 infection could not be derived from our data.

Our study has several limitations: (1) it was conducted at a single institution and the participants were non-immunocompromised, middle-aged adults; therefore, our findings might not be applicable to the general population; (2) because of a slight drop-out rate, anti-RBD antibodies and SARS-CoV-2-specific T cell response analyses were not available from all study subjects at all time points; (3) SARS-CoV-2 variants causing infections/reinfections in the study subjects were not characterized; instead, we relied on official data regarding circulating dominant variants during the study period; (4) variant-specific anti-RBD antibody determinations and neutralization studies were not performed; however, robust correlations between anti-RBD antibodies and viral neutralizing activity have been well established in other studies; and (5) analyses of the SARS-CoV-2-specific T cell response we performed were qualitative; therefore, changes in the magnitude of the cellular response over time could not be determined.

## 5. Conclusions

In healthy adults who received two doses of BNT162b2 followed by a booster of mRNA-273 and the bivalent Omicron-adapted BNT162b2 over a 26-month period, the evolution of anti-RBD antibodies suggests modulation of the immune response through immune imprinting. Waning of immune protection occurred, resulting in non-severe breakthrough infections in most study participants due to dominance of immune-evading variants in the presence of a durable vaccine-induced SARS-CoV-2-specific T cell response. These findings should be considered in the design of prospective studies oriented towards developing vaccination strategies against SARS-CoV-2.

## Figures and Tables

**Table 1:** SARS-CoV-2 infections and reinfections during follow-up.

		Months from 2nd Dose of the BNT162b2 Vaccine *								
	0	1.5	3	7	9	13	16	19	26	Total
N. of participants	268	258	256	255	238	224	203	216	166	-
SARS-CoV-2 infections (%)	37 (14)	13 (5)	1 (0.4)	14 (5.4)	8 (3.3)	72 (32)	54 (27)	83 (38)	63 (38)	345
Asymptomatic (%)	-	13 (100)	1 (100)	7 (50)	7 (87.5)	24 (33)	24 (44)	51 (61)	47 (75)	174 (50)
Symptomatic (%)	37 (100)	0	0	7 (50)	1 (12.5)	48 (67)	30 (56)	32 (38.5)	16 (25)	171 (50)
○ 1st infection (%)	37 (100)	13 (100)	1 (100)	11 (79)	4 (50)	51 (71)	38 (70)	39 (47)	23 (36.5)	217 (63)
○ Reinfection (%)	0	0	0	3 (21)	4 (50)	21 (29)	16 (30)	44 (53)	40 (63.5)	128 (37)

* All participants received two doses of BNT162b2. A booster dose of mRNA-1273 was administered to 248 (93%) study participants immediately after the 9-month time point; the bivalent Omicron-adapted BNT162b2 vaccine was administered to 93 (35%) study participants between the 19-month and the 26-month time points (see text for additional details).

**Table 2:** Subjects with SARS-CoV-2 breakthrough infections following the mRNA-1273 booster and bivalent Omicron-adapted BNT162b2 vaccine.

	mRNA-1273 Booster			Bivalent Omicron-Adapted BNT162b2		
SARS-CoV-2 breakthrough infection *	Yes *N* (%)	No *N* (%)	*p*-value **	Yes *N* (%)	No *N* (%)	*p*-value **
Yes	157 (67)	10 (83)	0.347	26 (36)	36 (39)	0.683
No	79 (33)	2 (17)		47 (64)	57 (61)	

* SARS-CoV-2 breakthrough infections were diagnosed as described in the Methods section (see text for details). ** Chi-squared test or Fisher’s exact test.

**Table 3:** Evolution of anti-RBD antibody titers in study participants.

	Estimated Anti-RBD Antibody Titers (AU/mL), GMT (95% CI)							
	Months from 2nd Dose of the BNT162b2 Vaccine ^a^							
	1.5	3	7	9	13	16	19	26
**All participants**	5384 (4556–6363)	4432 (3785–5165)	1325 * (1169–1503)	1305 (1151–1479)	13,966 * (11,962–16,906)	20,601 * (18,039–23,528)	20,214 (17,451–23,415)	17,040 (13,938–20,832)
**Age**								
**<47 years**	5869 (4811–7161)	4764 (3938–5764)	1473 * (1245–1742)	1223 (1019–1467)	12,581 * (10,371–15,262)	18,639 (15,457–22,476)	18,997 (15,558–23,195)	17,128 (13,328–22,013)
**≥47 years**	4931 (4023–6044)	4098 (3375–4975)	1153 * (973–1367)	857 (717–1026)	15,532 * (12,703–18,992)	22,812 (18,978–27,420)	21,534 (17,851–25,977)	16,950 (13,275–21,643)
**Gender**								
**Male**	5659 (4534–7064)	4734 (3817–5873)	1485 * (1221–1805)	1228 (998–1511)	16,068 * (12,767–20,223)	26,695 (21,539–33,086)	22,564 (18,183–28,000)	20,656 (15,671–27,229)
**Female**	5232 (4334–6314)	4250 (3558–5077)	1211 * (1040–1410)	924 (784–1089)	12,880 * (10,783–15,385)	17,740 (15,026–20,945)	18,972 (15,865–22,687)	15,249 (12,126–19,177)
**Previous SARS-CoV-2 infection ^b^**								
**No**	4570 (3828–5455)	3632 (3076–4289)	1076 (937–1236)	853 (734–991)	14,691 (12,411–17,389)	22,600 (19,526–26,158)	22,421 (19,188–26,200)	19,907 ** (16,105–24,608)
**Yes**	10,658 ** (8010–14,181)	10,028 ** (7587–13,254)	2909 ** (2230–3795)	2210 ** (1664–2933)	11,312 (8382–15,265)	14,009 (10,321–19,014)	13,128 (9447–18,244)	8916 (6205–12,810)
**SARS-CoV-2 infections during the study ^c^**								
**No**	5562 (4136–7478)	4528 (3362–6100)	1080 (815–1431)	808 (596–1097)	11,851 (8308–16,903)	14,001 (9247–21,199)	10,008 (6750–14,839)	8814 (5370–14,466)
**Yes**	5354 (4493–6380)	4403 (3737–5188)	1348 (1177–1543)	1068 (922–1237)	14,365 (12,217–16,891)	22,013 (19,539–26,619)	22,806 ** (19,539–26,619)	19,081 (15,445–23,573)

Abbreviations: Anti-RBD: antibodies against the receptor-binding domain of the S1 subunit of the spike protein of SARS-CoV-2; AU/mL: arbitrary units per milliliter; GMT, geometric mean titer; 95% CI: 95% confidence interval. ^a^ All participants received two doses of BNT162b2. A booster dose of mRNA-1273 was administered to 248 (93%) study participants immediately after the 9-month time point; the bivalent Omicron-adapted BNT162b2 vaccine was administered to 93 (35%) study participants between the 19-month and the 26-month time points. ^b^ Fifty (17%) participants had documented SARS-CoV-2 infections either before the first dose of BNT162b2 (*n* = 37) or prior to the 1.5-month time point (*n* = 13). ^c^ SARS-CoV-2 infections occurring before the first dose of BNT162b2, prior to the 1.5-month time point, or during follow-up; 53 (20%) subjects who were naïve at the 1.5-month time point remained uninfected throughout the entire study. * Significant difference (*p* < 0.05) compared with the previous time point. ** Significant difference (*p* < 0.05) among groups at indicated time points.

## Data Availability

The data presented in this study are available on request from the corresponding author.
